# Supplementary material for: A complete description of thermodynamic stabilities of molecular crystals
Source: Proc Natl Acad Sci U S A. 2022 Feb 7;119(6):e2111769119. doi: 10.1073/pnas.2111769119 (PMC8832981; doi:10.1073/pnas.2111769119)
Supplement: Supplementary File [file pnas.2111769119.sapp.pdf]

Supporting Information: A complete description of thermodynamic stabilities of molecular crystals

Venkat Kapil*

Yusuf Hamied Department of Chemistry, University of Cambridge, Lensfield Road, Cambridge, CB2 1EW,UK and Laboratory of Computational Science and Modeling, Institut des Matériaux, École Polytechnique Fédérale de Lausanne, 1015 Lausanne, Switzerland

Edgar A Engel†

TCM Group, Cavendish Laboratory, University of Cambridge, J. J. Thomson Avenue, Cambridge CB3 0HE, United Kingdom

(Dated: December 4, 2021)

* Correspondence email address: vk380@cam.ac.uk

† Correspondence email address: eae32@cam.ac.uk

structures	$1 \times 1 \times 1$ # of atoms	A_{MLP}	$2 \times 2 \times 2$ # of atoms	A_{MLP}
B-II - B-I	192	2.56672	1536	2.56993
α-G - γ-G	240	-1.19746	1920	-1.26223
β-G - γ-G	240	-1.26223	1920	-0.66375
α-S - β-S	336	-0.49998	2688	-0.48001

Table S1. Convergence of (harmonic) MLP free energies in kJ/mol with respect to simulation cell size.

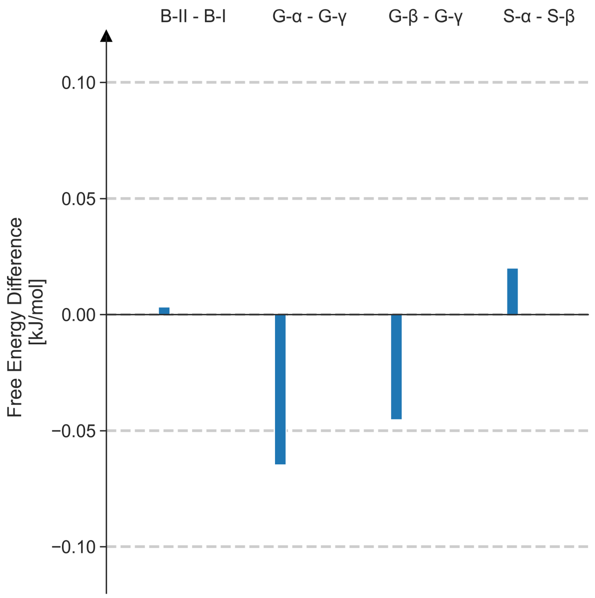

Figure S1. Convergence of (harmonic) MLP free energy difference between polymorphs with respect to simulation cell size.

S1. CONVERGENCE WITH SIMULATION CELL SIZE

To assess how converged the predicted free energy differences are with respect the sampling of the vibrational Brillouin zone (or conversely with respect to simulation cell size), we compute harmonic free energies for the simulation cells shown in Fig. 2 of the main text, as well as $2 \times 2 \times 2$ supercells of those simulation cells. The results are summarized in Table S1 and depicted in Fig. S1, and indicate that relative free energies for the supercells shown in Fig. 2 of the main text are converged to within 0.1 kJ/mol.

S2. CONSTRUCTION OF ROBUST MLPS

PI thermodynamic integration constitutes a practically universal method for computing free energies for a given potential. Thanks to the i-Pi code [1], which renders running harmonic approximations and PI molecular dynamics straight-forward, the streamlined formulation described in section ?? represents a simple computational task. Consequently, the universal applicability and user-friendliness of our protocol for computing rigorous free energies, hinges only on the ability to construct robust MLPs. Generating MLPs capable of stable PI simulations, especially in the fixed-pressure ensemble, in an automated fashion is non-trivial. This has motivated the development of efficient workflows for generating MLPs for specific state points such as Ref. [2]. Here we follow a simple strategy, which derives its efficiency and robustness from those of the 3ob parametrization [3] of DFTB theory [4] for organic molecular crystals.

In the main text, we demonstrate the efficiency and accuracy of our free energy protocol for benzene, glycine, and succinic acid. Here we complement this exposition by demonstrating the generation of robust MLPs for aspirin, paracetamol and a compound from the sixth blind test of organic crystal structure prediction methods [5]. For this purpose we restrict ourselves to DFTB rather than more costly PBE0-MBD reference data. We note that DFTB qualitatively reproduces the key features of the first-principles potential and free energy surfaces [6], including the quasi-free rotation of the methyl groups in paracetamol and aspirin, as well as conformations of molecular groups in XXIII [5].

To assess the resultant DFTB-based MLPs for aspirin, paracetamol, and compound XXIII from Ref. [5] for free energy calculations as outlined in the main text, we perform PI molecular dynamics simulations in the constant-pressure ensemble. We (i) confirm stability over at least 100 ps, (ii) compare the observed equilibrium finite-temperature cell parameters and volumes to the experimental values, and (iii) determine the free energy correction to the reference theory. The results are summarised in Table S2 and show that our approach allows rapid development of MLPs for industrially relevant molecular systems that can be used for rigorous free energy calculations.

form	RMSE energy	RMSE forces	volume [Å^3]	cell parameters a [Å]	b [Å]	c [Å]	α [°]	β [°]	γ [°]	FE correction [meV/atom]
B I	0.1	20.6	106 (112)	11.1 (11.3)	11.1 (11.3)	13.7 (14.1)	90 (90)	90 (90)	90 (90)	-0.1
B II	0.2	26.9	114 (107)	11.2 (11.2)	11.6 (10.9)	14.2 (14.4)	90 (90)	90 (90)	90 (90)	-0.5
α-G	0.4	84.5	78 (78)	15.2 (15.1)	12.4 (12.0)	10.0 (10.2)	90 (90)	85 (84)	90 (90)	-0.3
β-G	0.5	93.9	78 (79)	15.1 (15.3)	12.6 (12.5)	9.9 (9.9)	90 (90)	84 (85)	90 (90)	-0.3
γ-G	0.6	84.8	79 (78)	14.2 (14.0)	12.3 (12.1)	10.8 (10.9)	90 (90)	90 (90)	90 (90)	-1.2
α-S	0.1	49.4	123 (128)	11.0 (10.9)	17.0 (17.5)	16.5 (16.3)	100 (95)	101 (101)	93 (92)	0.3
β-S	0.1	40.2	119 (120)	10.9 (10.9)	17.7 (17.5)	14.8 (15.0)	90 (90)	93 (93)	90 (90)	-0.9
A I	1.04	89.2	208 (209)	13.5 (12.1)	4.6 (6.5)	13.60 (11.4)	90 (90)	99 (111)	90 (90)	0.7
A II	1.33	86.2	209 (213)	14.4 (11.4)	4.5 (6.6)	13.70 (11.4)	90 (90)	109 (95)	80 (90)	1.5
P I	1.72	85.8	100 (97)	13.5 (12.9)	8.8 (9.4)	7.2 (7.1)	91 (90)	113 (115)	90 (90)	-0.9
P II	1.16	86.5	97 (94)	12.4 (11.8)	17.2 (17.2)	7.3 (7.4)	90 (90)	90.0 (90)	97 (90)	-1.0
XXIII	5.0	120.0	1933 (1892)	12.7 (13.1)	10.5 (10.5)	14.3 (16.2)	90 (90)	106 (105)	90 (90)	0.35

Table S2. Overview of MLP performance for polymorphs of benzene (B-I and B-II), glycine (α-G, β-G, γ-G), succinic acid (α-S and β-S), aspirin (A-I and A-II)m paracetamol (P-I and P-II), and a polymorph of a blind test compound (XXIII). The energy and force RMSEs in meV/atom and meV/ Å, the ensemble average cell parameters and cell volumes, and the free energy (FE) correction from the MLP to reference theory level, are obtained from simulations for the supercells (shown in Fig. 2 of the main text for the first three compounds). All data for benzene, glycine, and succinic acid is based on and compared to PBE0-MBD reference data, while that for aspirin, paracetamol and XXIII is based on and compared to DFTB data (as proof-of-principle). For comparison, the experimental values from Refs. [7–12] are given in parenthesis. The fairly good agreement of the DFTB-based cell parameters for the latter three compounds highlights the versatility of the DFTB parametrization of choice for organic molecular crystals.

---

[1] V. Kapil, M. Rossi, O. Marsalek, R. Petraglia, Y. Litman, T. Spura, B. Cheng, A. Cuzzocrea, R. H. Meißner, D. M.Wilkins, B. A. Helfrecht, P. Juda, S. P. Bienvenue, W. Fang, J. Kessler, I. Poltavsky, S. Vandenbrande, J. Wieme, and M. Ceriotti, Computer Physics Communications

236, 214 (2018).

[2] C. Schran, F. L. Thiemann, P. Rowe, E. A. Müller, O. Marsalek, and A. Michaelides, Proceedings of the National Academy of Sciences **118**, e2110077118 (2021).

[3] T. Krüger, M. Elstner, P. Schiffels, and T. Frauenheim, The Journal of Chemical Physics **122**, 114110 (2005).

[4] "DFTB+, a software package for efficient approximate density functional theory based atomistic simulations: The Journal of Chemical Physics: Vol 152, No 12,".

[5] A. M. Reilly, R. I. Cooper, C. S. Adjiman, S. Bhattacharya, A. D. Boese, J. G. Brandenburg, P. J. Bygrave, R. Bylsma, J. E. Campbell, R. Car, D. H. Case, R. Chadha, J. C. Cole, K. Cosburn, H. M. Cuppen, F. Curtis, G. M. Day, R. A. DiStasio Jr, A. Dzyabchenko, B. P. van Eijck, D. M. Elking, J. A. van den Ende, J. C. Facelli, M. B. Ferraro, L. Fusti-Molnar, C.-A. Gatsiou, T. S. Gee, R. de Gelder, L. M. Ghiringhelli, H. Goto, S. Grimme, R. Guo, D. W. M. Hofmann, J. Hoja, R. K. Hylton, L. Iuzzolino, W. Jankiewicz, D. T. de Jong, J. Kendrick, N. J. J. de Klerk, H.-Y. Ko, L. N. Kuleshova, X. Li, S. Lohani, F. J. J. Leusen, A. M. Lund, J. Lv, Y. Ma, N. Marom, A. E. Masunov, P. McCabe, D. P. McMahon, H. Meekes, M. P. Metz, A. J. Misquitta, S. Mohamed, B. Monserrat, R. J. Needs, M. A. Neumann, J. Nyman, S. Obata, H. Oberhofer, A. R. Oganov, A. M. Orendt, G. I. Pagola, C. C. Pantelides, C. J. Pickard, R. Podeszwa, L. S. Price, S. L. Price, A. Pulido, M. G. Read, K. Reuter, E. Schneider, C. Schober, G. P. Shields, P. Singh, I. J. Sugden, K. Szalewicz, C. R. Taylor, A. Tkatchenko, M. E. Tuckerman, F. Vacarro, M. Vasileiadis, A. Vazquez-Mayagoitia, L. Vogt, Y. Wang, R. E. Watson, G. A. de Wijs, J. Yang, Q. Zhu, and C. R. Groom, Acta Crystallographica Section B: Structural Science, Crystal Engineering and Materials **72**, 439 (2016).

[6] M. Rossi, P. Gasparotto, and M. Ceriotti, Physical Review Letters **117**, 115702 (2016).

[7] A. Katrusiak, M. Podsiadło, and A. Budzianowski, Crystal Growth and Design **10**, 3461 (2010).

[8] A. Dawson, D. R. Allan, S. A. Belmonte, S. J. Clark, W. I. F. David, P. A. McGregor, S. Parsons, C. R. Pulham, and L. Sawyer, Crystal Growth and Design **5**, 1415 (2005).

[9] J.-L. Leviel, G. Auvert, and J.-M. Savariault, Acta Crystallographica **B37**, 2185 (1981).

[10] E. J. Chan, T. R. Welberry, A. P. Heerdegen, and D. J. Goossens, Acta Cryst. B **66**, 696 (2010).

[11] M. Haisa, S. Kashino, R. Kawai, and H. Maeda, Acta Cryst. B **32**, 1283 (1976).

[12] M. Haisa, S. Kashino, and H. Maeda, Acta Cryst. B **30**, 2510 (1974).
